# Understanding climate engagement and open recognition in European higher education: A mixed-methods study across four countries

**DOI:** 10.12688/openreseurope.19909.1

**Published:** 2025-05-06

**Authors:** Pablo Martín-Ramos, Adriana Correa-Guimaraes, Fatma Fourati-Jamoussi, Kimberley Burcke-Couchy, Lucio Alessandro Lo Giudice, Barbara Tosi, Frederico Oliveira Pinto, Luís Veiga Martins, Luis Manuel Navas-Gracia

**Affiliations:** 1 ETSIIAA, University of Valladolid, Avenida de Madrid 44, Palencia, 34004, Spain; 2 InTerACT (UP 2018.C102), UniLaSalle, 19 Rue Pierre Waguet, Beauvais, 60000, France; 3 Consorzio Scuola Comunità Impresa (CSCI), Via Francesco Sesalli 7, Novara, 28100, Italy; 4 School of Business and Economics, NOVA University, R. da Holanda 1, Carcavelos, 2775-405, Norway

**Keywords:** Digital credentials, Educational policy, Environmental competencies, European Green Deal, Informal learning, Micro-credentials, Sustainability literacy, Value-action gap

## Abstract

**Background:**

This mixed-methods study investigates student engagement with climate issues and perceptions of open recognition systems across four European educational institutions in France, Italy, Portugal, and Spain as part of the OpenPass4Climate Erasmus+ project. Against the backdrop of the European Green Deal and UNESCO's call for transformative education, our research addresses the critical need for innovative climate education approaches that bridge knowledge and action.

**Methods:**

Through a comprehensive approach combining surveys ( *n*=630), individual interviews ( *n*=69), national focus groups ( *n*=45), and a transnational focus group ( *n*=16), we examined students' climate attitudes, educational preferences, and views on digital badge systems for recognizing climate competencies.

**Results:**

Results reveal a notable disconnect between strong climate concern (mean=4.0/5) and moderate personal responsibility (3.2/5), alongside significant cross-country variations in environmental engagement, with Portuguese students consistently demonstrating the highest climate awareness and Italian students the lowest. While respondents strongly endorsed formal climate curriculum integration (4.1/5) and valued informal learning pathways (3.8/5), they reported limited participation in eco-pedagogical activities (2.3/5), highlighting an implementation gap in environmental education. Students rated their informal climate change education (3.5/5) more highly than formal training (3.2/5), suggesting untapped potential for recognition of non-formal learning experiences. Gender differences emerged consistently, with female respondents showing significantly higher environmental concern and engagement across multiple dimensions. Analysis of open badge perceptions revealed moderate familiarity but substantial interest, particularly when aligned with institutional credentialing systems and employer recognition frameworks.

**Conclusions:**

Key implementation challenges identified include the need for robust quality assurance mechanisms, institutional endorsement, and enhanced digital infrastructure accessibility. These findings inform strategic recommendations for developing the European Open Badges Passport, emphasizing the importance of balancing standardization with contextual flexibility while facilitating recognition of both formal and informal climate learning across diverse higher education settings.

## Plain language summary

Our research investigated how university students across four European countries (France, Italy, Portugal, and Spain) think about and engage with climate change issues, and what they think about using digital badges to recognize climate-related learning. We collected information through an online survey of 630 students, individual interviews with 69 participants, group discussions in each country, and an international group discussion.

The study revealed important insights about students' perspectives on climate change and environmental education. While students expressed strong concern about climate change, they felt only moderately responsible for taking action themselves. We found notable differences across demographics and countries: female students and those in advanced degree programs showed higher environmental awareness, and Portuguese students consistently demonstrated the highest climate awareness, while Italian students showed the lowest. Although students valued both formal education and informal learning about climate issues, they reported low participation in actual environmental activities. When it came to recognition of climate-related learning through digital badges, students showed moderate interest but expressed concerns about how employers would view these credentials.

These findings are significant because they help us understand how to better engage students in climate education and action. The research provides valuable insights for developing the European Open Badges Passport, a system that will recognize both formal and informal climate learning achievements. Our results suggest that effective climate education needs to bridge the gap between awareness and action, while recognition systems must balance standardization across Europe with flexibility for local needs. The findings emphasize the importance of ensuring digital badges are credible and valued by employers, while making climate education more accessible through both traditional and innovative learning approaches. These insights will directly inform how European universities approach climate education and recognize students' environmental competencies in the coming years.

## Abstract (es)

**Antecedentes:**

Este estudio de métodos mixtos investiga la implicación de los estudiantes con los problemas climáticos y las percepciones de los sistemas de reconocimiento abierto en cuatro instituciones educativas europeas en Francia, Italia, Portugal y España como parte del proyecto Erasmus+ OpenPass4Climate. En el contexto del Pacto Verde Europeo y el llamamiento de la UNESCO para una educación transformadora, nuestra investigación aborda la necesidad crítica de enfoques innovadores de educación climática que vinculen el conocimiento y la acción.

**Métodos:**

A través de un enfoque integral que combina encuestas (n=630), entrevistas individuales (n=69), grupos de discusión nacionales (n=45) y un grupo de discusión transnacional (n=16), examinamos las actitudes de los estudiantes hacia el clima, sus preferencias educativas y sus opiniones sobre los sistemas de insignias digitales para el reconocimiento de competencias climáticas.

**Resultados:**

Los resultados revelan una notable desconexión entre una fuerte preocupación por el clima (media=4,0/5) y una responsabilidad personal moderada (3,2/5), junto con variaciones significativas entre países en el compromiso ambiental, mostrando los estudiantes portugueses sistemáticamente la mayor concienciación climática y los estudiantes italianos la menor. Aunque los encuestados respaldaron firmemente la integración formal del clima en el plan de estudios (4,1/5) y valoraron las vías de aprendizaje informal (3,8/5), informaron de una participación limitada en actividades eco-pedagógicas (2,3/5), lo que pone de manifiesto una brecha de implementación en la educación ambiental. Los estudiantes valoraron más su educación informal sobre el cambio climático (3,5/5) que su formación formal (3,2/5), lo que sugiere un potencial sin explotar para el reconocimiento de experiencias de aprendizaje no formal. Las diferencias de género surgieron de manera consistente, mostrando las encuestadas una preocupación y compromiso ambiental significativamente mayores en múltiples dimensiones. El análisis de las percepciones sobre las insignias abiertas reveló una familiaridad moderada pero un interés sustancial, particularmente cuando están alineadas con los sistemas de acreditación institucional y los marcos de reconocimiento de los empleadores.

**Conclusiones:**

Los principales desafíos de implementación identificados incluyen la necesidad de mecanismos sólidos de garantía de calidad, respaldo institucional y mejora de la accesibilidad a la infraestructura digital. Estos hallazgos informan recomendaciones estratégicas para el desarrollo del Pasaporte Europeo de Insignias Abiertas, enfatizando la importancia de equilibrar la estandarización con la flexibilidad contextual, facilitando al mismo tiempo el reconocimiento del aprendizaje climático tanto formal como informal en diversos entornos de educación superior.

## Abstract (fr)

**Contexte:**

Cette étude à méthodes mixtes examine l'engagement des étudiants envers les questions climatiques et leurs perceptions des systèmes de reconnaissance ouverts dans quatre établissements d'enseignement européens en France, Italie, Portugal et Espagne, dans le cadre du projet Erasmus+ OpenPass4Climate. Dans le contexte du Pacte vert pour l'Europe et de l'appel de l'UNESCO à une éducation transformatrice, notre recherche répond au besoin crucial d'approches innovantes en matière d'éducation au climat qui établissent un pont entre les connaissances et l'action.

**Méthodes:**

Grâce à une approche complète combinant des enquêtes (n=630), des entretiens individuels (n=69), des groupes de discussion nationaux (n=45) et un groupe de discussion transnational (n=16), nous avons examiné les attitudes des étudiants face au climat, leurs préférences éducatives et leurs opinions sur les systèmes de badges numériques pour la reconnaissance des compétences climatiques.

**Résultats:**

Les résultats révèlent un décalage notable entre une forte préoccupation climatique (moyenne=4,0/5) et une responsabilité personnelle modérée (3,2/5), ainsi que des variations significatives entre les pays en matière d'engagement environnemental, les étudiants portugais démontrant systématiquement la plus grande sensibilisation au climat et les étudiants italiens la plus faible. Bien que les répondants aient fortement approuvé l'intégration formelle du climat dans les programmes d'études (4,1/5) et valorisé les parcours d'apprentissage informels (3,8/5), ils ont signalé une participation limitée aux activités éco-pédagogiques (2,3/5), mettant en évidence un écart de mise en œuvre dans l'éducation environnementale. Les étudiants ont évalué leur éducation informelle sur le changement climatique (3,5/5) plus favorablement que leur formation formelle (3,2/5), suggérant un potentiel inexploité pour la reconnaissance des expériences d'apprentissage non formelles. Des différences de genre ont émergé de façon constante, les répondantes montrant une préoccupation et un engagement environnementaux significativement plus élevés dans de multiples dimensions. L'analyse des perceptions des badges ouverts a révélé une familiarité modérée mais un intérêt substantiel, particulièrement lorsqu'ils sont alignés avec les systèmes d'accréditation institutionnels et les cadres de reconnaissance des employeurs.

**Conclusions:**

Les principaux défis de mise en œuvre identifiés comprennent la nécessité de mécanismes d'assurance qualité robustes, d'approbation institutionnelle et d'amélioration de l'accessibilité des infrastructures numériques. Ces résultats éclairent les recommandations stratégiques pour le développement du Passeport Européen des Badges Ouverts, soulignant l'importance d'équilibrer la standardisation avec la flexibilité contextuelle tout en facilitant la reconnaissance des apprentissages climatiques formels et informels dans divers contextes d'enseignement supérieur.

## Abstract (de)

**Hintergrund:**

Diese Mixed-Methods-Studie untersucht das Engagement von Studierenden für Klimafragen und ihre Wahrnehmung offener Anerkennungssysteme an vier europäischen Bildungseinrichtungen in Frankreich, Italien, Portugal und Spanien im Rahmen des Erasmus+-Projekts OpenPass4Climate. Vor dem Hintergrund des europäischen Green Deals und des UNESCO-Aufrufs zu transformativer Bildung befasst sich unsere Forschung mit dem kritischen Bedarf an innovativen Ansätzen zur Klimabildung, die Wissen und Handeln miteinander verbinden.

**Methoden:**

Durch einen umfassenden Ansatz, der Umfragen (n=630), Einzelinterviews (n=69), nationale Fokusgruppen (n=45) und eine transnationale Fokusgruppe (n=16) kombiniert, untersuchten wir die Einstellungen der Studierenden zum Klima, ihre Bildungspräferenzen und ihre Ansichten zu digitalen Badge-Systemen zur Anerkennung von Klimakompetenzen.

**Ergebnisse:**

Die Ergebnisse zeigen eine bemerkenswerte Diskrepanz zwischen starker Klimabesorgnis (Mittelwert=4,0/5) und mäßiger persönlicher Verantwortung (3,2/5) sowie signifikante länderübergreifende Unterschiede im Umweltengagement, wobei portugiesische Studierende durchweg das höchste Klimabewusstsein und italienische Studierende das niedrigste aufweisen. Während die Befragten die formale Integration des Klimas in die Lehrpläne (4,1/5) nachdrücklich befürworteten und informelle Lernwege schätzten (3,8/5), berichteten sie von einer begrenzten Teilnahme an ökopädagogischen Aktivitäten (2,3/5), was eine Umsetzungslücke in der Umweltbildung verdeutlicht. Die Studierenden bewerteten ihre informelle Klimawandelbildung (3,5/5) höher als ihre formale Ausbildung (3,2/5), was auf ein unausgeschöpftes Potenzial für die Anerkennung nicht-formaler Lernerfahrungen hindeutet. Geschlechterunterschiede traten konsequent zutage, wobei weibliche Befragte in mehreren Dimensionen ein signifikant höheres Umweltbewusstsein und Engagement zeigten. Die Analyse der Wahrnehmung offener Badges ergab eine moderate Vertrautheit, aber ein erhebliches Interesse, insbesondere wenn sie mit institutionellen Zertifizierungssystemen und Anerkennungsrahmen von Arbeitgebern in Einklang gebracht werden.

**Schlussfolgerungen:**

Zu den identifizierten Hauptherausforderungen bei der Umsetzung gehören die Notwendigkeit robuster Qualitätssicherungsmechanismen, institutioneller Unterstützung und verbesserter Zugänglichkeit digitaler Infrastrukturen. Diese Erkenntnisse liefern strategische Empfehlungen für die Entwicklung des Europäischen Open Badges Passes, wobei die Bedeutung einer ausgewogenen Standardisierung mit kontextueller Flexibilität hervorgehoben wird, während gleichzeitig die Anerkennung von formalem und informellem Klimaelernen in verschiedenen Hochschulkontexten erleichtert wird.

## Abstract (it)

**Contesto:**

Questo studio a metodi misti indaga il coinvolgimento degli studenti nelle questioni climatiche e le percezioni dei sistemi di riconoscimento aperto in quattro istituzioni educative europee in Francia, Italia, Portogallo e Spagna come parte del progetto Erasmus+ OpenPass4Climate. Nel contesto del Green Deal europeo e dell'appello dell'UNESCO per un'educazione trasformativa, la nostra ricerca affronta la necessità critica di approcci innovativi all'educazione climatica che colmino il divario tra conoscenza e azione.

**Metodi:**

Attraverso un approccio completo che combina sondaggi (n=630), interviste individuali (n=69), focus group nazionali (n=45) e un focus group transnazionale (n=16), abbiamo esaminato gli atteggiamenti degli studenti verso il clima, le loro preferenze educative e le loro opinioni sui sistemi di badge digitali per il riconoscimento delle competenze climatiche.

**Risultati:**

I risultati rivelano una notevole disconnessione tra una forte preoccupazione per il clima (media=4,0/5) e una moderata responsabilità personale (3,2/5), insieme a significative variazioni tra i paesi nell'impegno ambientale, con gli studenti portoghesi che dimostrano costantemente la più alta consapevolezza climatica e gli studenti italiani la più bassa. Mentre i rispondenti hanno fortemente sostenuto l'integrazione formale del clima nei programmi di studio (4,1/5) e hanno valorizzato i percorsi di apprendimento informale (3,8/5), hanno riportato una partecipazione limitata alle attività eco-pedagogiche (2,3/5), evidenziando un divario di implementazione nell'educazione ambientale. Gli studenti hanno valutato la loro educazione informale sul cambiamento climatico (3,5/5) più positivamente della formazione formale (3,2/5), suggerendo un potenziale non sfruttato per il riconoscimento delle esperienze di apprendimento non formale. Le differenze di genere sono emerse costantemente, con le rispondenti femmine che mostrano una preoccupazione e un impegno ambientale significativamente più elevati in più dimensioni. L'analisi delle percezioni dei badge aperti ha rivelato una familiarità moderata ma un interesse sostanziale, particolarmente quando allineati con i sistemi di accreditamento istituzionale e i quadri di riconoscimento dei datori di lavoro.

**Conclusioni:**

Le principali sfide di implementazione identificate includono la necessità di solidi meccanismi di garanzia della qualità, supporto istituzionale e una migliore accessibilità dell'infrastruttura digitale. Questi risultati informano raccomandazioni strategiche per lo sviluppo del Passaporto Europeo dei Badge Aperti, sottolineando l'importanza di bilanciare la standardizzazione con la flessibilità contestuale, facilitando al contempo il riconoscimento dell'apprendimento climatico sia formale che informale in diversi contesti di istruzione superiore.

## Introduction

The fight against climate change is a cornerstone of the EU Erasmus+ program, with education recognized as a vital tool in confronting this global challenge ( European Commission, 2021a). The environmental crisis confronting humanity today transcends scientific boundaries, encompassing profound social and political dimensions ( Vare & Scott, 2007). As we approach the mid-2020s, the urgency of climate change and the associated loss of biodiversity necessitate an educational response, yet many schools and systems grapple with effective strategies ( UNESCO, 2021). Research consistently shows that merely imparting knowledge about environmental issues is insufficient to spur meaningful change; shifts in attitudes and perceptions of the natural world are essential to catalyzing behavioral transformation ( Ajzen, 1991; Bamberg & Möser, 2007; Kollmuss & Agyeman, 2002).

The European Union has responded decisively through initiatives such as the European Green Deal, launched in 2019, which outlines a roadmap for sustainable development ( European Commission, 2019). Moreover, its commitment has been further reinforced by policy recommendations that stress the importance of education for environmental sustainability and advocate for incorporating youth perspectives in tackling climate and biodiversity challenges—most notably, the Council Recommendation on Learning for the Green Transition and Sustainable Development ( Council of the European Union, 2022a) and the Council of Europe Recommendation on Young People and Climate Action ( Council of Europe, 2024). Complementing these efforts, the European Commission’s GreenComp framework provides a structured approach to fostering sustainability competencies across educational contexts ( Bianchi *et al.*, 2022).

Within this framework, the OpenPass4Climate project emerges as an innovative initiative under the Erasmus+ program, introducing an open recognition alliance system to enhance climate education. By leveraging Open Badges and the OpenPass4Climate platform, the project aims to elevate climate-related activities from awareness to actionable commitment and justice. It seeks to evaluate the tangible impacts of these efforts on advancing climate justice and to identify mechanisms that accelerate the adoption of positive behaviors. A core objective is the creation of a lifelong, portable OpenPass4Climate system, empowering individuals to engage actively in climate initiatives.

To ensure the system’s efficacy, WP3 focused on understanding students’ engagement with climate issues, assessing key climate commitments, and exploring perceptions of both implicit and explicit recognition mechanisms. This comprehensive study spanned four European countries —France, Italy, Portugal, and Spain— and employed multiple research methods: an online survey, one-to-one interviews, national focus groups, and a transnational focus group. These efforts provide insights into youth perceptions of climate change, educational preferences, and attitudes toward open recognition systems, informing the co-design of an effective Open Badge system.

This article details the research methodology, presents findings from all study components, analyzes results within the context of contemporary European environmental and educational frameworks, and offers recommendations for the OpenPass4Climate system’s implementation. The findings aim to support the development of student-centered curricula and flexible learning pathways that bridge knowledge and action, fostering a generation of environmentally conscious citizens.

## Methods

This study adopted a mixed-methods design, integrating quantitative and qualitative approaches across four phases: an online survey, one-to-one interviews, national focus groups, and a transnational focus group. This multifaceted strategy ensured both broad data collection and in-depth exploration of participants’ perspectives on climate education and open recognition systems.

### Survey design and implementation

A 20-question survey (Annex I in the Extended Data) was administered to students from four European countries (France, Italy, Portugal, and Spain). The survey, designed by consortium members and crafted in compliance with the European Union's General Data Protection Regulation, collected demographic information including age, gender, education level, and country of study. Gender was determined through self-identification, with participants selecting from options including 'Male', 'Female', 'Non-binary', 'Prefer not to say', and an open text option for other gender identities. This approach aligns with best practices in gender-inclusive research design.

The survey investigated seven key constructs: *‘Climate change views’* examined students' acknowledgment of scientific evidence attributing climate change to human activity and their perception of it as a significant threat. ‘ *Environmental values and identity’* explored the importance students attribute to environmental protection and their self-identification as environmentally friendly ( Bogner & Wiseman, 2006; Johnson & Manoli, 2010). ‘ *Personal responsibility and emotional responses’* investigated students' sense of responsibility for mitigating climate change effects and their confidence in making a positive impact. ‘ *Social norms’* explored perceptions of others' expectations and behaviors regarding environmental issues. ‘ *Eco-pedagogical activities’* investigated prior formal and informal climate education. ‘ *Learning interests and training preferences’* assessed interest in climate change learning and preferred approaches. The ‘ *Open badges system/awareness’* examined familiarity with open badges for recognizing climate-related achievements.

Responses were provided on a Likert scale (1–5), with extreme scale values explicitly defined for all questions.

The survey instrument was initially pilot tested with a group of students from CSCI to assess clarity, comprehensibility, and response patterns. Feedback from this pilot phase was incorporated into the final version of the questionnaire. Internal consistency and reliability were assessed using Cronbach's alpha coefficient.

### One-to-one interviews

Interview participants were selected from survey respondents who had indicated willingness to participate in follow-up research and provided contact information after reviewing data protection consent information. The final sample comprised 69 participants: 17 from France (UniLaSalle Beauvais), 23 from Italy (CSCI Novara), 10 from Portugal (Nova School of Business and Economics), and 19 from Spain (Universidad de Valladolid).

A set of 12 questions (Annex III in the Extended Data), designed by consortium members based on feedback from each partner institution’s teaching staff and students, structured the interviews into four blocks: *‘Climate change views’*, ‘ *Eco-pedagogical activities’*, ‘ *Learning interests and training preferences’*, and ‘ *Open badges system/awareness’*. Interviews were conducted in participants' native languages without time constraints, allowing respondents to elaborate as needed for each question.

### National focus groups

National focus groups were conducted in each participating country with a total of 45 participants: 17 French undergraduate students, 6 Italian vocational and high-school students, 12 Portuguese students across different levels, and 10 Spanish undergraduate and Master-level students. The focus groups addressed 12 questions (Annex IV in the Extended Data) organized into four thematic blocks: ‘ *Climate change views’*, ‘ *Personal responsibility and social norms’*, ‘ *Learning interests and training preferences’*, and ‘ *Open badges system/awareness*’.

Each consortium member conducted the focus group in their country using the participants' native language. As in the case of the one-to-one interviews, to comply with GDPR requirements, personally identifiable information and full transcripts were only accessible to the educational institution to which students belonged, with anonymized reports shared among consortium members.

### International focus group

The transnational focus group involved 16 students, four from each country, selected to ensure diverse educational representation: Italian high school/vocational students, French undergraduates, Portuguese Master's students, and Spanish PhD-level students. The session was conducted using Miro as the online meeting platform, chosen for its EU GDPR compliance.

Participants were contacted by their institutions regarding purpose, objectives, and expected outcomes, with informed consent obtained for participation and research data use. Rather than recording the session, Miro's notes feature captured relevant information and insights during discussions, facilitating free and open dialogue while maintaining participant privacy.

### Data analysis

The analysis followed a sequential mixed-methods approach. Quantitative survey data underwent statistical analysis using IBM SPSS Statistics v.27 (IBM Corp., Armonk, NY, USA). Given that assumptions of normality were not met, the Kruskal-Wallis non‐parametric test was employed to determine statistically significant differences among groups. *Post hoc* multiple pairwise comparisons used the Conover-Iman test. For reproducibility purposes, all statistical analyses conducted in this study can be replicated using R statistical software, an open-source alternative that provides equivalent statistical capabilities. Qualitative data from interviews and focus groups were subjected to thematic analysis. The integration phase employed a convergent design, wherein quantitative and qualitative findings were compared and contrasted to identify areas of convergence and divergence.

To address potential methodological limitations, several measures were implemented. The sample's composition, while diverse, showed some inherent limitations that were accounted for in the analysis. Categories with limited sample sizes were excluded from the summary of survey results. Furthermore, since age and educational level categories substantially overlapped, only the more detailed educational level data was retained in the analysis.

Translation and interpretation across multiple languages required careful attention. While interviews and focus groups were conducted in participants' native languages to ensure authentic expression, subsequent translation of findings into English for cross-consortium analysis involved review by multiple consortium members fluent in both the source language and English to maintain the accuracy and consistency of findings.

## Results

### Survey findings

***Reliability and sample characteristics***

**Figure 1.**
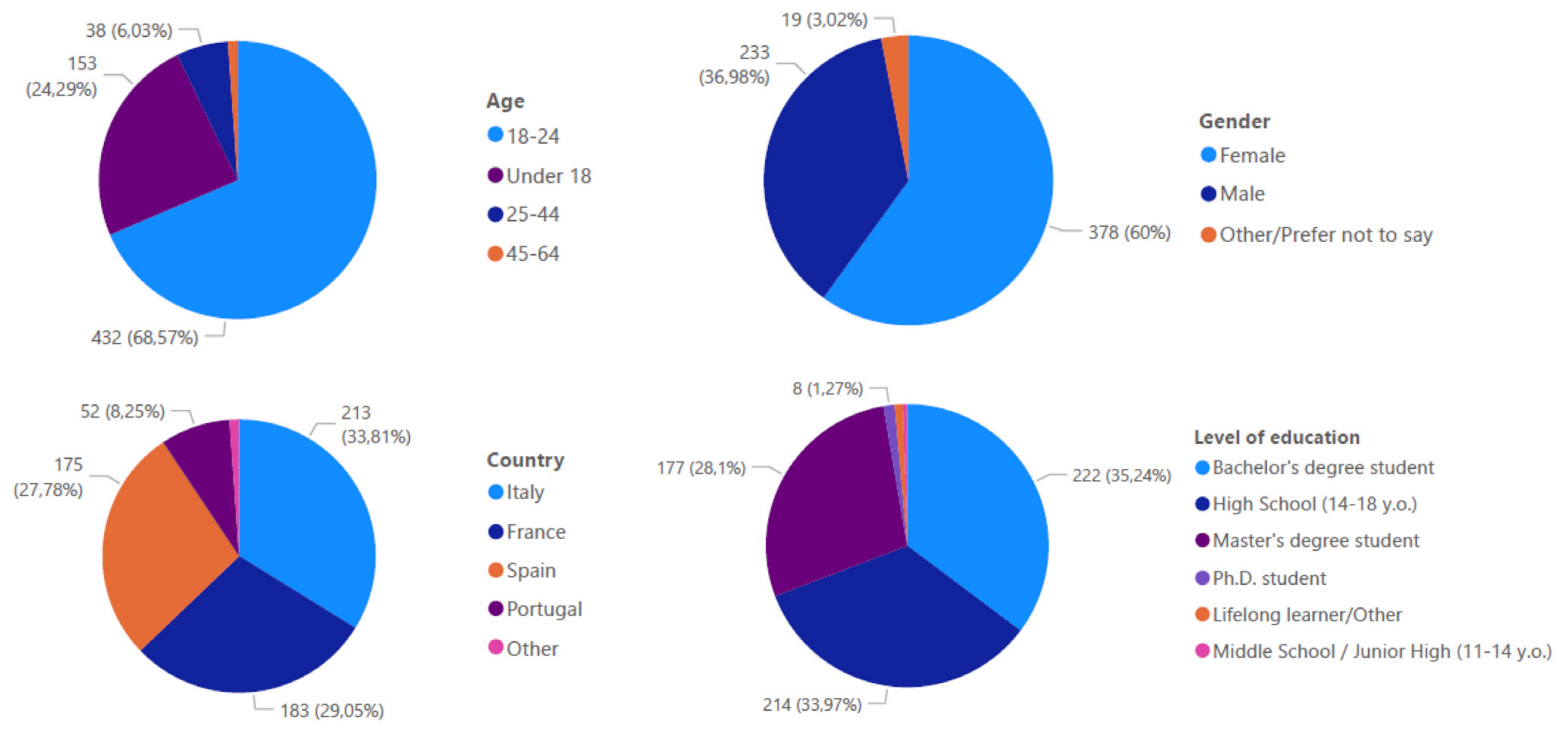
Profile of the survey respondents.

The survey garnered responses from 630 participants, with demographic analysis ( Figure 1) revealing that 69% fell within the 18–24 age bracket. Gender distribution showed 60% female respondents compared to 37% male respondents. The sample's educational composition included Bachelor's degree students (35%, with 150 from Spain, 43 from France, and 27 from Portugal), A-level students (34%, predominantly from Italy), and Master's degree students (28%, with 134 from France, 19 from Portugal, and 18 from Spain).

The survey's internal consistency, assessed through Cronbach's alpha coefficient (0.847) and standardized Cronbach's alpha (0.848), demonstrated strong reliability in measuring latent factors among subjects.

***Climate attitudes and values***

*Climate change views (questions 1–4; Annex II in the Extended Data, Tables S1 and S2)*

Respondents expressed significant concern about climate change (mean=4.0/5), with strong consensus acknowledging human activities as major contributors (4.4/5). Participants generally believed in an individual capacity to impact climate change (3.7/5) and strongly endorsed government intervention (4.4/5). Statistical analysis revealed:

- Significantly higher scores among female students across all four questions
- Higher concern levels among university students compared to other educational levels
- Country-level variations with Italian students showing the lowest scores and Portuguese students consistently highest

*Environmental values and identity (questions 5–7; Annex II in the Extended Data, Tables S1 and S2)*

Environmental protection received high importance ratings (4.5/5), though prioritizing it over economic growth garnered less support (3.8/5). Respondents reported medium-high alignment between actions and environmental values (3.6/5), with notable variations:

- Female students showed the highest scores for environmental values items
- No significant gender differences in action alignment
- Master's students and French students reported the lowest alignment
- Portuguese students demonstrated the highest alignment scores

*Personal responsibility and emotional responses (questions 8–9; Annex II in the Extended Data, Tables S1 and S2)*

Results showed a moderate sense of responsibility for reducing climate change effects (3.2/5), with:

- Significantly higher scores among female respondents
- Lowest scores among French students
- Highest scores among Portuguese students
- General lack of hope about the environmental future (2.6/5)
- Greater optimism among the youngest respondents and Portuguese/Italian students

*Social norms (questions 10–11; Annex II in the Extended Data, Tables S1 and S2)*

Participants reported medium levels of environmental concern in their social circles (2.9/5), with:

- Highest scores among Master's students
- Lower perceptions (2.4/5) of environmental care by authority figures
- Lowest scores in France
- Highest scores in Italy and Portugal

***Educational engagement***

*Eco-pedagogical activities (questions 12–14; Annex II in the Extended Data, Tables S3 and S4)*

Formal climate change training was rated as moderate (3.2/5), while informal education scored slightly higher (3.5/5). Key findings included:

- Higher scores among female and Master's students
- Higher participation rates in France and Portugal
- Low overall participation in eco-pedagogical activities (2.3/5)

*Learning interests and training preferences (questions 15–17; Annex II in the Extended Data, Tables S3 and S4)*

Students showed moderate-to-high interest in climate-related learning (3.8/5), with:

- Strong support for formal curriculum integration (4.1/5)
- High endorsement of informal learning effectiveness (4.1/5)
- Higher scores among female and Master's level students
- Particularly strong interest in France and Portugal

***Open recognition systems***

*Open badges system/awareness (questions 18–20; Annex II in the Extended Data, Tables S3 and S4)*

Results indicated:

- Moderate valuation of climate-related learning recognition (3.5/5)
- Limited motivation for earning open badges (3.2/5)
- Neutral stance on employability impact (3.0/5)
- Higher optimism among Portuguese students (3.5/5)

Statistical analysis revealed significant country-level differences for 12 out of 20 items, emphasizing the importance of considering national contexts in implementation strategies.

### Interview and focus group findings

***One-to-one interviews***

*Participant profiles*

The interviews involved 69 participants across four countries:

- France: 17 respondents (14 females, 3 males), primarily second-year Environmental Engineering and Agri-Food degree students
- Italy: 23 respondents (14 males, 9 females) aged 17–18, representing diverse educational fields including Administration, Finance, Marketing, Agriculture, and others
- Portugal: 10 Master-level students (5 females, 5 males) from the School of Business and Economics
- Spain: 19 respondents (11 females, 8 males) across undergraduate (11), Master's level (3), and PhD-level (5) programs

*Common themes across countries*

Pressing environmental issues emerged as a primary concern, with climate change universally recognized as the most critical issue. Participants specifically highlighted global warming, CO _2_ emissions, pollution across multiple domains (air, water, soil), deforestation, biodiversity loss, and waste management challenges.

Individual actions were emphasized across all interviews, with participants identifying key behavioral changes such as reducing meat consumption, using public transportation, practicing recycling, raising awareness through education, and adopting responsible consumption habits.

Participants across countries supported regulatory measures and environmental taxes to drive change, emphasizing the role of politicians, governments, and corporations. Most respondents reported receiving climate-related training through both formal university courses and informal methods such as workshops and documentaries.

*Country-specific insights*

Italian participants provided detailed insights into local waste management practices and emphasized the need for improved energy efficiency systems. French students demonstrated a strong interest in innovative sustainability solutions and emphasized the importance of robust government policies. Portuguese respondents highlighted community-driven initiatives and advocated for comprehensive educational reforms. Spanish participants showed clear preferences for experiential and practical learning methods, with a strong dislike for purely online learning formats.

***National focus groups***

*Participant distribution*

The national focus groups comprised:

- France: 17 undergraduate students (14 females, 3 males)
- Italy: 6 vocational and high-school students (3 females, 3 males)
- Portugal: 12 students (7 females, 5 males) across Bachelor's (5), Master's (5) and PhD (2) levels
- Spain: 10 undergraduate and Master-level students (8 females, 2 males)

*Key findings*

Participants across all countries acknowledged the severity and urgency of climate change, citing issues such as global warming, extreme weather events, pollution, and biodiversity loss. There was strong consensus regarding climate change's impact on future generations, particularly concerning food security, health issues, economic instability, and increased migration.

Education emerged as crucial in fostering environmental awareness and promoting sustainable practices. Participants advocated for integrating environmental education into school curricula from an early age, ensuring comprehensive approaches beyond superficial treatment of topics.

Country-specific variations revealed:

- Italian participants emphasized economic consequences, particularly agricultural impacts
- French participants discussed eco-anxiety and psychological effects
- Portuguese participants focused on greenwashing issues and corporate transparency
- Spanish participants provided detailed insights into local climate impacts

### International focus group

The transnational focus group involved 16 students (4 per country) representing different educational levels. Discussions focused on badge design, implementation preferences, and potential concerns.

***Badge design and comprehension***

Participants critiqued the badge design, noting readability challenges with white text on light backgrounds. They suggested green and orange hues to reflect nature and confidence, respectively, alongside clearer classification descriptions and refined graphics and logo sizes for visual clarity.

***Implementation recommendations***

For implementation, they advocated user-friendly features, such as progress-tracking tools and detailed tutorials, as well as highlighting training hours in metadata for EU-wide recognition. They also proposed gamification (e.g., weekly challenges) and integration with LinkedIn and university transcripts to enhance utility.

***Potential concerns***

Participants raised concerns about employer preferences for traditional certificates, uncertainty about badge value in job applications, and risks of superficial learning focused on badge acquisition rather than genuine knowledge gain. They emphasized the need for technical support and guidance for organizations implementing the badge system.

These findings provided insights for developing the badge system's design and implementation strategy while highlighting important considerations for ensuring its effectiveness and credibility.

## Discussion

The multi-method approach of this study provides robust insights into youth climate attitudes, educational preferences, and perceptions of open recognition systems. Building on prior European research, such as the Special Eurobarometer 501 ( European Commission, 2020a) and 538 ( European Commission, 2023), the findings reveal both encouraging trends and persistent challenges in climate education and competency development, particularly within educational contexts and among younger populations.

### Environmental attitudes and climate awareness

Concerning the first construct on ' *Climate change views*', a comparison of our survey results with the Eurobarometer findings reveals important insights into youth climate attitudes within the broader European context. Our student respondents demonstrated high climate concern (Q1, mean=4.0/5), which aligns with but intensifies the general public sentiment captured in Eurobarometer 538 (QC2, *'How serious a problem do you think climate change is at this moment?*'), where 77% of EU citizens view climate change as a very serious problem. Country-specific trends (Portugal, 4.48 > France, 4.11 > Spain, 3.91 > Italy, 3.76) were quite consistent with those obtained in the Eurobarometer 538 (Portugal, 89% > Spain, 86% > France, 85% > Italy, 83%), with Portugal and Italy ranking first and last, respectively.

The OpenPass4Climate survey (Q2) provided unique insight into students' strong belief in human responsibility for climate change (4.4/5 overall, with Portugal's highest at 4.8/5), a perspective not directly measured in the Eurobarometer surveys.

When examining individual roles in tackling climate change (Q3), student respondents showed a stronger belief in individual impact (3.7/5) compared to the relatively low percentages in Eurobarometer 538 (QC3, ' *In your opinion, who within the EU is responsible for tackling climate change?*'), in which only 35% of respondents (EU27-average) felt personally responsible for tackling climate change. This suggests that students may feel more empowered than the general population. Interestingly, Portugal showed the highest belief in our survey (3.98/5), and lower than the EU27 average (28%) in the Eurobarometer, while Italy showed the lowest belief in both surveys (3.57/5 and 20%, respectively).

Students showed strong support for government intervention (Q4, 4.4/5) in tackling climate change. This aligns with the aforementioned Eurobarometer 538 (QC3) responses, in which national governments and the EU were chosen by 56% of the respondents as responsible for tackling climate change. This also connects with strong support for coordinated intervention, with Eurobarometer 501 (QA8) showing that 70% of the respondents favor joint EU decision-making on environmental issues. As for country differences, Portuguese students' strong support in our survey (4.63/5) contrasts with the lower support in Eurobarometer 538's QC3 (47% and 52% for national governments and the EU, respectively), while Italy's lowest scores in both surveys are consistent.

With regard to the ‘ *Environmental values and identity*’ construct, the relationship between environmental values and actions revealed interesting patterns across surveys. Younger populations demonstrated heightened endorsement of environmental protection (Q5, 4.5/5) compared to national averages: in Eurobarometer 501 (QA1, ‘ *How important is protecting the environment to you personally?*’), only 53% —EU-27 average value— of the respondents chose ‘very important’. The highest score among Portuguese students (4.75/5) aligns with the results from the TIMSS 2023 survey where Portugal ranked better (522±2.6) than Italy (517±3.7) and France (511±4.3) in terms of ‘Students Value Environmental Preservation’ ( von Davier *et al.*, 2024).

However, despite strong endorsement of environmental protection, alignment between values and actions was moderate (Q7, 3.6/5), highlighting a ‘value-action gap’ ( Uitto *et al.*, 2015). Focus groups further elucidated this gap, pointing to structural and institutional barriers, a phenomenon well-documented in environmental psychology ( Kollmuss & Agyeman, 2002). This disconnect finds parallels in Eurobarometer 538 (QC5, *‘Have you personally taken any action to fight climate change over the past six months?’*) where only 63% of the respondents said ‘yes’, with concrete actions varying significantly (QC6), and with Eurobarometer 501 (QA9), in which 67% of the respondents recognized that —as citizens— they were not doing enough to protect the environment. Italy’s highest action-value alignment in our survey (3.71/5) contrasts with its lowest value in Eurobarometer 538 (52%), suggesting that in this country the gap may be reduced among more environmentally aware youth populations.

For the ‘ *Personal responsibility and emotional responses*’ construct, a dimension not covered in the Eurobarometer surveys, our survey shows students felt only moderately responsible for reducing the negative effects of climate change (Q8, 3.2/5), with a lower value than the one indicated above for Q3 on how much individuals can make a difference in addressing climate change (3.7/5). This further supports that they predominantly place the onus for climate action on governments while underestimating potential individual responsibility and contributions. Regarding the hopefulness about the future of the environment (Q9), they were mostly pessimistic (2.6/5). This prevailing sense of hopelessness about the environment's future is consistent with their perception of those in leadership positions within their countries, which they consider can make a difference, as indifferent to environmental issues (see below).

Analysis of the ‘ *Social norms*’ construct reveals that respondents in our survey considered that people in their social circle cared less than them about the environment and climate change (Q10, 2.9/10 vs. Q5, 4.5/5), which does not align with the actual perception in their countries according to Eurobarometer 538 QC2 (83 to 89% of respondents considered climate change a very serious problem). Their view on how much people in positions of responsibility (national government, regional government) in their country care and take action to protect the environment and climate change yielded a very low value (Q11, 2.3/5). This aligns with Eurobarometer 538 (QC7), in which 67% (EU27-average) of the respondents believed their governments are not doing enough, and with Eurobarometer 501 (QA9), in which 72% and 68% of the respondents considered that their national governments and the EU were not doing enough to protect the environment.

As for other aspects not analyzed in the Eurobarometer surveys, gender differences emerged in our survey, with female respondents consistently scoring higher on environmental values, aligning with established research ( McCright, 2010; Zelezny *et al.*, 2000), though Portugal's uniformly high scores suggest cultural and educational contexts may mitigate such disparities.

The educational level also emerged as a significant factor in environmental engagement. Master's students showed the highest rates of climate awareness and advocacy, which aligns with UNESCO's analysis of how specialized education enhances pro-environmental behavior, particularly among STEM graduates ( Nair-Bedouelle, 2021).

### Eco-pedagogical activities, learning interests, and training preferences

Results from the ' *Eco-pedagogical activities*' construct indicate that students rated their informal climate change education (Q13, 3.5/5) more highly than their formal training (Q12, 3.2/5). This preference for informal learning sources mirrors Eurobarometer 501's QA4 findings, where respondents primarily relied on television news (66%) and the internet (38%) for environmental information rather than formal educational channels. The higher scores for perceived climate change training in France (Q12, 3.51/5; Q13, 3.66/5), with Portugal ranking second, do not align with the results from the TIMSS 2023 survey where Portugal ranked better (520±2.9) than Italy (504±3.8) and France (492±3.6) in terms of ‘Average Environmental Knowledge’ score ( von Davier *et al.*, 2024).

Despite high interest in climate-related learning (Q15, 3.8/5), participation in eco-pedagogical activities was low (Q14, 2.3/5). This participation gap mirrors the findings from Eurobarometer 501, in which eco-pedagogical activities (e.g., attending a workshop about environmental issues or a collective beach or park cleanup) showed consistently low engagement rates across member states (7% EU-27 average). Moreover, in Eurobarometer 501’s QA10, providing more information and education was only chosen as an effective way of tackling environmental issues by 24% of the respondents. Our student survey suggests a higher valuation of educational interventions among university students compared to the general population, highlighting the potential role of higher education in fostering environmental engagement, and that barriers remain to convert students’ strong interest in climate education into active participation in educational initiatives.

On the topic of training preferences, participants strongly favored hybrid learning models, valuing formal curriculum integration (Q16, 4.1/5) and informal pathways (Q17, 3.8/5). National focus groups underscored the complementary roles of structured and experiential learning, with students advocating for a balance that combines theoretical knowledge with practical, community-based activities. This preference aligns with the European Commission’s emphasis on flexible learning pathways ( European Commission, 2021a) and the GreenComp framework’s call for holistic sustainability education that fosters critical thinking, problem-solving, and collaboration ( Bianchi *et al.*, 2022).

### Open recognition systems: bridging theory and practice

The identified value-action gap suggests that recognition systems could incentivize action, even though the initial survey showed that students only moderately valued recognition for completing climate-related learning activities (Q18, 3.5/5) and showed even less interest in open badges as a motivation tool to learn about climate-related topics (Q19, 3.2/5). As subsequently evidenced during the one-to-one interviews and national focus groups, this was partly due to the students’ limited understanding of the open badges system and its value for recognizing and showcasing climate-related learning achievements (underscoring the need for targeted awareness campaigns and educational initiatives regarding its purpose and applications) and partly due to real-world applicability concerns, a notion supported by the transnational focus group’s emphasis on quality assurance, institutional credibility, and alignment with existing qualification frameworks. Participants expressed concerns about employer recognition of badges, technological barriers (e.g., lack of access to digital platforms in rural areas), and the risk of superficial learning if badges are awarded without rigorous assessment. These challenges resonate with broader EU competency framework efforts ( European Commission: Joint Research Centre, 2025) and UNESCO’s call for transformative education that fosters deep, meaningful engagement ( UNESCO, 2021).

On the question of employability (Q20, 3/5), the survey's neutral perception contrasts with evidence from the World Economic Forum's 2023 Future of Jobs Report, which indicates growing employer recognition of sustainability-focused credentials ( Di Battista *et al.*, 2023). This discrepancy aligns with critiques of poor institutional communication about credential portability ( Oliver, 2022). However, Portuguese students were more optimistic (3.5/5), which may be tentatively attributed to Portugal’s comprehensive digital education strategy, particularly the Digital Transition Action Plan (PDE) adopted in 2020, which the European Schoolnet highlighted as a transformative System Change Case Study ( Wastiau *et al.*, 2024). This initiative, complemented by the National Digital Skills Initiative e.2030 (INCoDe.2030) and the Portuguese government's strategic funding of Universidade Aberta, has created an integrated approach to digital micro-credentials and open education for certifying competencies ( Griffiths *et al.*, 2024). Such coordinated national efforts align with and advance broader EU initiatives to embed Open Badges in educational frameworks ( Council of the European Union, 2022b; EQAR, 2023).

Addressing these concerns will require careful design, ensuring that badges are credible, user-friendly, and integrated with existing educational and professional frameworks. For example, badges could be aligned with the European Qualifications Framework ( European Commission, 2018) to enhance their recognition across borders. The European Council’s emphasis on whole-institution approaches ( Council of the European Union, 2022a) provides a supportive context for such developments, encouraging universities to adopt open recognition systems as part of their sustainability strategies.

### Policy implications

The research findings advocate for coordinated policy interventions across European, national, and institutional levels to advance climate education and open recognition systems. Each level presents distinct challenges and opportunities that must be addressed through carefully crafted policy frameworks.

At the European level, the European Union must establish comprehensive frameworks that balance standardization with national adaptability. This requires developing quality assurance mechanisms for open badge systems that set minimum standards while allowing flexibility for national and regional adaptation ( Council of the European Union, 2022b). These mechanisms should ensure alignment with existing European qualification frameworks ( European Commission, 2018) while supporting accessibility through multilingual resources and platforms. The EU must also provide funding for digital infrastructure, particularly in underserved regions, and establish standardized protocols for badge portability across member states. Integration with broader EU initiatives is crucial, including alignment with European Green Deal educational objectives ( European Commission, 2019) and coordination with the Digital Education Action Plan implementation to support cross-border recognition of climate-related credentials.

National-level policy development should focus on the systemic integration of climate education and recognition within existing educational frameworks. Curriculum development requires thoughtful integration of climate competencies into national frameworks, with consideration given to increasing mandatory climate education hours ( UNESCO, 2022). Additionally, assessment frameworks must evolve to recognize both formal and informal learning pathways. Teacher preparation represents another crucial area for national policy intervention, as highlighted by Redman *et al.* (2018). Countries should establish systematic professional development programs and create support networks for teaching staff, providing necessary resources for eco-pedagogical activities. Recognition systems at the national level must create clear pathways for validating informal learning and establish mechanisms for incorporating open badges into national qualification systems, with quality assurance processes aligned with EU standards ( European Commission: Joint Research Centre, 2025).

At the institutional level, educational organizations require policy support for implementing comprehensive approaches to climate education and recognition ( Breiting *et al.*, 2005; Higgs & McMillan, 2006; Leal-Filho *et al.*, 2021; Mathar, 2015). Organizational strategy should embrace whole-institution approaches to climate education while establishing partnerships with environmental organizations ( UNESCO, 2021). Institutions must develop clear policies for recognizing external learning and integrating sustainability across academic programs. Infrastructure development demands significant attention, with investments needed in digital platforms for badge issuance and verification, alongside technical support systems for users. Quality assurance at the institutional level requires robust assessment criteria and verification processes for badge issuance, supported by careful documentation of learning outcomes.

These policy recommendations align with key EU frameworks, including the European Skills Agenda's emphasis on transversal skills recognition ( European Commission, 2020b), the Digital Education Action Plan's promotion of innovative learning tools ( European Commission, 2021b), and the European Green Deal's focus on environmental sustainability education ( European Commission, 2019). Successful implementation requires careful attention to several critical factors. Policymakers must maintain a delicate balance between standardization and contextual flexibility while ensuring equitable access to recognition systems. Support for technological infrastructure development must be coupled with building credibility through robust quality assurance, such as the European Blockchain Service Infrastructure (EBSI). Furthermore, policies should foster collaboration between educational institutions and industry to enhance the value and recognition of climate-related credentials.

Implementation should follow an evidence-based approach while remaining adaptable to emerging needs and technological developments in digital credentialing. The findings from this study suggest that effective policy frameworks must address both the technical aspects of open badge systems and the broader educational and social contexts in which they operate ( Bianchi *et al.*, 2022). As climate education evolves and digital recognition systems mature, policies must remain responsive to changing needs while maintaining high standards for quality and accessibility.

### Limitations and future research directions

This study has several limitations that future research should address. The sample's skew toward higher education students may limit generalizability to other educational contexts. Additionally, the rapid evolution of digital credentialing systems means that attitudes toward open badges may shift as these tools become more widespread. Future research should explore:

–    Longitudinal impacts of open recognition systems on learning outcomes

–    Comparative effectiveness of different badge implementation strategies

–    Intersection of climate competencies with other key EU competency frameworks

–    Role of emerging technologies, like blockchain, in credential verification to enhance security and trust

Our findings underscore the complex interplay between environmental attitudes, educational preferences, and recognition systems in supporting climate competency development. Success will require carefully coordinated efforts across policy domains and stakeholder groups, with particular attention to maintaining a balance between standardization and contextual flexibility.

## Conclusions

The OpenPass4Climate research reveals critical insights for developing effective climate education and open recognition systems in European higher education. The study across four countries demonstrates that while students show strong climate awareness, there remains a significant gap between knowledge and action. This gap presents both a challenge and an opportunity for the development of open badge systems.

The findings suggest three key areas requiring immediate attention for the successful implementation of the OpenPass4Climate initiative:

First, educational systems must bridge formal and informal learning pathways. Our research demonstrates that students value both structured academic approaches and experiential learning opportunities, suggesting that open badge systems should recognize and validate both forms of learning.

Second, badge system design must prioritize credibility and usability. The focus group findings emphasize that successful implementation requires clear institutional backing, robust quality assurance mechanisms, and user-friendly interfaces. These elements are essential for ensuring that badges hold value for both academic progression and professional development.

Third, implementation strategies must account for national and cultural contexts while maintaining European-wide standards. The significant variations in climate attitudes and educational preferences across countries indicate the need for flexible frameworks that can adapt to local contexts while ensuring consistent quality and recognition.

Looking ahead, the OpenPass4Climate initiative must work within existing European educational frameworks while pushing boundaries to create innovative recognition systems. The focus should be on developing badges that not only acknowledge learning but actively encourage engagement with climate issues through practical action.

The path forward requires a coordinated effort from educational institutions, policymakers, and stakeholders to create meaningful change in climate education. Success will depend on maintaining the delicate balance between standardization and flexibility, ensuring that open badges serve as effective tools for recognizing and promoting climate competency development across Europe.

## Ethics and consent

This research was conducted in accordance with the ethical principles of the Declaration of Helsinki. The study was approved by the Technical Directorate for Privacy Matters, with approval from the Data Protection Officer of the University of Valladolid (Reference Number: UVA/26/2023) on September 21, 2023. The study was conducted in accordance with the University of Valladolid's Ethical Code, approved by the Governing Council on July 22, 2022.

Informed consent was obtained from all participants through the online survey platform, where participants were informed that by taking the survey they indicated they had read and understood the data protection and consent statement and agreed to participate. For focus groups and interviews, participants provided verbal consent after reviewing data protection information. All participants were informed of data confidentiality measures and their rights under GDPR requirements, including the right to withdraw from the study at any time without penalty. Data were anonymized during analysis and reporting to protect participant identities.

## Data Availability

UVaDOC Documentary Repository of the University of Valladolid: Understanding climate engagement and open recognition in European higher education: A mixed-methods study across four countries. https://doi.org/10.35376/10324/75208 This project contains the following underlying data: –    OpenPass4Climate_survey_results.ods (Complete dataset of survey responses from 630 participants across four European countries). UVaDOC Documentary Repository of the University of Valladolid: Understanding climate engagement and open recognition in European higher education: A mixed-methods study across four countries. https://uvadoc.uva.es/handle/10324/75250 This project contains the following extended data: –    Annex I: Survey questionnaire –    Annex II: Statistical analysis tables including mean Likert scale scores, Kruskal-Wallis test results (mean of ranks) and Conover-Iman multiple pairwise comparisons –    Annex III: One-to-one interview questionnaire –    Annex IV: National focus group questionnaire All data are available under the terms of the Creative Commons Zero "No rights reserved" data waiver (CC0 Public domain dedication). This study follows the Standards for Reporting Qualitative Research (SRQR) guidelines for the qualitative components and the Strengthening the Reporting of Observational Studies in Epidemiology (STROBE) guidelines for the survey component. Additionally, the study adheres to the Sex and Gender Equity in Research (SAGER) guidelines for reporting sex and gender information.
