# Supplementary material for: Understanding climate engagement and open recognition in European higher education: A mixed-methods study across four countries
Source: Open Res Eur. 2025 Aug 28;5:121. Originally published 2025 May 6. [Version 2] doi: 10.12688/openreseurope.19909.2 (PMC12529058; doi:10.12688/openreseurope.19909.2)
Supplement: Peer review history

# Reviewer report: Reviewer response for version 2

**DOI:** 10.21956/openreseurope.22908.r61692

**Published:** 2025-11-08

**Authors:** Jennie Stephens

**Affiliations:** 1 National University of Ireland Maynooth, Co. Kildare, Ireland

The literature review section entitled "climate education in higher education" is insufficient and does not adequately reflect the breadth of prior research on climate education in higher education" including for example: 

 Kinol, A., et al. (2023). "Climate justice in higher education: a proposed paradigm shift towards a transformative role for colleges and universities." Climatic Change 176(2): 15.

 In addition, this section should at least make a nod to acknowledging the broader sustainability education literature that also include climate education.  By narrowly focusing only on climate education the authors risk perpetuating climate isolationism which is the phenomenon when people focus narrowly on climate without acknowledging how the climate crisis is intricately connected with larger problems of ecological destruction and growing vulnerabilities of human well-being

If applicable, is the statistical analysis and its interpretation appropriate?

I cannot comment. A qualified statistician is required.

Is the study design appropriate and is the work technically sound?

Yes

Is the work clearly and accurately presented and does it engage with the current literature?

Partly

Are the conclusions drawn adequately supported by the results?

Yes

Are sufficient details of methods and analysis provided to allow replication by others?

Yes

Are all the source data and materials underlying the results available?

Yes

Reviewer Expertise:

Climate justice in higher education.

I confirm that I have read this submission and believe that I have an appropriate level of expertise to confirm that it is of an acceptable scientific standard.

# Reviewer report: Reviewer response for version 2

**DOI:** 10.21956/openreseurope.22908.r60483

**Published:** 2025-10-15

**Authors:** Ihtsham Ul Haq Padda

**Affiliations:** 1 Federal Urdu University of Arts, Science and Technology, Islamabad, Pakistan

The introduction of the article is well written and provides a clear overview of the research context. However, the significance of the study is not sufficiently articulated in connection with the contemporary global challenges of climate change and the urgent need to build resilience within higher education systems. Strengthening this linkage would enhance the relevance and impact of the study.

 The literature review is adequate and provides a general background to the topic. Nonetheless, it appears somewhat limited, particularly concerning the dimension of climate change awareness and engagement practices within educational institutions. Expanding this section to include more recent and diverse sources would enrich the theoretical foundation.

 The methodology is sound, following standard research procedures, and has been implemented in a well-organized manner. The management of the study is commendable, and the results are clearly presented with robust empirical evidence. However, the discussion section could be improved by incorporating more analytical depth—interpreting the findings beyond numerical presentation to offer stronger insights and implications.

 In conclusion, this is a well-conducted and valuable study that contributes meaningfully to the growing body of literature on climate change and education. With minor revisions—particularly in highlighting the study’s significance and enhancing the analytical discussion—it can make an important scholarly contribution to the field.

If applicable, is the statistical analysis and its interpretation appropriate?

Yes

Is the study design appropriate and is the work technically sound?

Yes

Is the work clearly and accurately presented and does it engage with the current literature?

Yes

Are the conclusions drawn adequately supported by the results?

Yes

Are sufficient details of methods and analysis provided to allow replication by others?

Yes

Are all the source data and materials underlying the results available?

Yes

Reviewer Expertise:

Development Economics | Public Sector Economics | Environmental Economics | Food Security | Climate Change

I confirm that I have read this submission and believe that I have an appropriate level of expertise to confirm that it is of an acceptable scientific standard.

# Reviewer report: Reviewer response for version 2

**DOI:** 10.21956/openreseurope.22908.r61100

**Published:** 2025-10-15

**Authors:** Gaye Defne Ceyhan

**Affiliations:** 1 Bogazici University, Istanbul, Turkey

This study investigates climate engagement and open recognition systems across four European countries. The findings present a valuable contribution to the field of climate education and digital credentialing. The revised manuscript successfully addresses reviewer concerns through substantial enhancements to the introduction, literature review, methods section, and discussion.

 The addition of the literature review section and enhanced theoretical justification for survey constructs strengthens the manuscript's scholarly positioning. The cross-national design with multiple data collection methods offers valuable comparative insights into climate education needs and cultural variations.

 The revised manuscript includes detailed methodological explanations, explicit acknowledgment of limitations, and full data availability, supporting reproducibility and further research. The findings provide actionable recommendations for implementing open badge systems in climate education contexts. The authors have appropriately acknowledged the limitations of the study and proposed future research directions.

 The revised manuscript is clearly written, methodologically sound, and provides valuable insights for researchers, educators, and policymakers working on climate education and recognition systems in European higher education. After reviewing the revised manuscript, I am pleased to recommend its acceptance.

If applicable, is the statistical analysis and its interpretation appropriate?

Yes

Is the study design appropriate and is the work technically sound?

Yes

Is the work clearly and accurately presented and does it engage with the current literature?

Yes

Are the conclusions drawn adequately supported by the results?

Yes

Are sufficient details of methods and analysis provided to allow replication by others?

Yes

Are all the source data and materials underlying the results available?

Yes

Reviewer Expertise:

Climate change education, sustainability education, systems thinking

I confirm that I have read this submission and believe that I have an appropriate level of expertise to confirm that it is of an acceptable scientific standard.

# Reviewer report: Reviewer response for version 2

**DOI:** 10.21956/openreseurope.22908.r59195

**Published:** 2025-10-01

**Authors:** Anh Thu Nguyen

**Affiliations:** 1 Mount Royal University, Calgary, Canada

After reviewing the revised manuscript, we are pleased to recommend its acceptance. We appreciate the thoroughness with which the authors addressed our constructive comments, particularly their treatment of the limitation concerning the lack of collective political actions. The manuscript is now highly accessible, with a clear and logical flow. As a result, the revisions have successfully strengthened the introduction and the literature review, clarified the methodology, and enhanced both the discussion and conclusions.

 We commend the authors for their careful and thoughtful revisions, which have substantially improved the clarity and overall quality of the manuscript.

If applicable, is the statistical analysis and its interpretation appropriate?

Yes

Is the study design appropriate and is the work technically sound?

Partly

Is the work clearly and accurately presented and does it engage with the current literature?

Partly

Are the conclusions drawn adequately supported by the results?

Yes

Are sufficient details of methods and analysis provided to allow replication by others?

Partly

Are all the source data and materials underlying the results available?

Yes

Reviewer Expertise:

Sustainable consumption, sustainability education, climate change education

I confirm that I have read this submission and believe that I have an appropriate level of expertise to confirm that it is of an acceptable scientific standard.

# Reviewer report: Reviewer response for version 1

**DOI:** 10.21956/openreseurope.21536.r53980

**Published:** 2025-07-24

**Authors:** Anh Thu Nguyen, Paul Berger

**Affiliations:** 1 Mount Royal University, Calgary, Canada; 2 Lakehead University, Thunder Bay,, Canada

Thank you for the opportunity to review the manuscript. It touches a very important topic relating to climate change education with a study across four countries in Europe.

1. The introduction clearly sets a context for the research. We strongly recommend adding a literature review section to provide a current landscape of research on the topics and to identify research gaps that justify this research. Also, an outline of the paper structure could be provided at the end of the introduction for clarity. Please write acronyms out in full on their first usage. 
2. Methods: The research design is appropriate; however, we suggest stronger justifications as to why this research design was used. The authors could explain more clearly the choice of research methods with support from theories on research methodologies. Additionally, the authors could elaborate on the choice of seven key constructs used in the survey design.
3. More details need to be provided about data collection methods for replication by others and to understand potential biases. For instance, how the authors conducted surveys in four countries (online, email, paper-based, etc), how they recruited students (university and high-school students), and how they recruited participants for the transnational focus group (what were the selection criteria?)
4. The analysis is good, with results reported clearly. However, we strongly recommend the authors to clearly explain the measures used to overcome methodological limitations; for example, what are some inherent limitations in the sample’s composition and how they were accounted for in the analysis. What categories had limited sample sizes? Please specify them. Quantitative results are typically reported with more detail, such as the effect size and confidence level; is there a reason why your results omitted details? Perhaps these are in Annex II.
5. The discussion could be improved by providing a more robust synthesis with the existing literature. As a literature review is not provided, it is difficult for the reader to see ‘significant’ findings, in comparison with what is known in literature. Some discussion is needed to help readers understand how Likert averages can be compared to percentages in the Eurobarometer findings and to speculate on comparisons between student and general population results.

References to the Eurobarometer findings are needed.

1. It is unclear why political action is not highlighted in this study; why emphasise individual actions such as eating less meat and asking about government responsibility, without asking whether participants have taken part in climate protests, signed petitions or contacted elected officials?

Thank you again for the opportunity to review the manuscript. We hope our comments help improve the work. We wish the authors the best of luck with the research!

If applicable, is the statistical analysis and its interpretation appropriate?

Yes

Is the study design appropriate and is the work technically sound?

Partly

Is the work clearly and accurately presented and does it engage with the current literature?

Partly

Are the conclusions drawn adequately supported by the results?

Yes

Are sufficient details of methods and analysis provided to allow replication by others?

Partly

Are all the source data and materials underlying the results available?

Yes

Reviewer Expertise:

Sustainable consumption, sustainability education, climate change education

We confirm that we have read this submission and believe that we have an appropriate level of expertise to confirm that it is of an acceptable scientific standard, however we have significant reservations, as outlined above.

# Response

**Published:** 2025-08-09

**Authors:** Pablo Martín-Ramos

**Affiliations:** Ingeniería Agrícola y Forestal, Universidad de Valladolid, Palencia, Castile and León, Spain

Thank you for the opportunity to review the manuscript. It touches a very important topic relating to climate change education with a study across four countries in Europe.

***Response**: Thank you for your thorough and constructive review of our manuscript. We appreciate the interdisciplinary perspectives you bring from sustainability education and climate change pedagogy. We have carefully addressed each of your comments with substantial revisions to strengthen the manuscript. Below, we provide a detailed point-by-point response with specific changes made.*

**Reviewer comment 1**: The introduction clearly sets a context for the research. We strongly recommend adding a literature review section to provide a current landscape of research on the topics and to identify research gaps that justify this research. Also, an outline of the paper structure could be provided at the end of the introduction for clarity. Please write acronyms out in full on their first usage. 

***Response**: We appreciate this suggestion and have added a new Literature Review section following the Introduction. As this manuscript is part of the OpenPass4Climate project, which includes a comprehensive scoping review published separately, we provide a focused synthesis that highlights key gaps our empirical study addresses while directing readers to the full review for detailed analysis.*

*Changes made:*

*(1) A new paragraph providing an outline of the paper structure has been added at the end of the Introduction.*

*(2) A new section 2 (Literature review) has been added after the Introduction.*

*(3) “European Union (EU)", "United Nations Educational, Scientific and Cultural Organization (UNESCO)", "Science, Technology, Engineering, and Mathematics (STEM)”, "General Data Protection Regulation (GDPR)", and "Trends in International Mathematics and Science Study (TIMSS)" are now written in full at first use.*

**Reviewer comment 2**: Methods: The research design is appropriate; however, we suggest stronger justifications as to why this research design was used. The authors could explain more clearly the choice of research methods with support from theories on research methodologies. Additionally,the authors could elaborate on the choice of seven key constructs used in the survey design.

***Response**: We have substantially expanded the Methods section to provide theoretical justification for our methodological choices and elaborate on the survey constructs' theoretical foundations.*

*Changes made:*

*(1) The first paragraph of the Methods section has been rewritten, and a new subsection on “Research design and rationale” has been added.*

*(2) The original “Survey design and implementation” subsection has been divided into two subsections. The survey design has been expanded to a new “Survey design and theoretical framework” subsection.*

**Reviewer comment 3**: More details need to be provided about data collection methods for replication by others and to understand potential biases. For instance, how the authors conducted surveys in four countries (online, email, paper-based, etc), how they recruited students (university and high-school students), and how they recruited participants for the transnational focus group (what were the selection criteria?)

***Response**: We have comprehensively revised the data collection section to provide full operational details, enabling replication and bias assessment.*

*Changes made: The original “Survey design and implementation” subsection has been divided into two subsections. The survey implementation has been expanded to a new “Data collection procedures” section, with “Survey administration, Interview recruitment and selection”, and Focus group recruitment” subsection.*

**Reviewer comment 4**: The analysis is good, with results reported clearly. However, we strongly recommend the authors to clearly explain the measures used to overcome methodological limitations; for example, what are some inherent limitations in the sample’s composition and how they were accounted for in the analysis. What categories had limited sample sizes? Please specify them. Quantitative results are typically reported with more detail, such as the effect size and confidence level; is there a reason why your results omitted details? Perhaps these are in Annex II.

***Response**: We appreciate the reviewers' attention to methodological rigor and statistical reporting. We have expanded our discussion of limitations and clarified our statistical approach.*

*Regarding effect sizes and confidence intervals: We acknowledge that these measures are not included in Annex II. Our analytical approach employed non-parametric methods (Kruskal-Wallis tests with Conover-Iman post-hoc comparisons) appropriate for ordinal Likert data that violated normality assumptions. For such non-parametric analyses, effect size measures are less standardized than for parametric tests. The mean ranks and rank differences provided in Tables S2 and S4 serve an analogous interpretive function: larger rank separations indicate stronger practical effects. For example, the 145.6-point rank difference between Italy and Portugal on climate concern (mean ranks: 260.0 vs. 405.6, p<.0001) clearly indicates a substantial effect beyond mere statistical significance.*

*Confidence intervals, typically calculated assuming interval data properties, would be inappropriate for our rank-based analyses of ordinal data. However, recognizing that some researchers may wish to apply parametric interpretations, we have made our complete dataset available in the repository, enabling alternative analytical approaches, including effect size calculations or confidence interval estimation, for those who consider Likert data to possess interval properties.*

*Changes made:*

*(1) The “Data analysis” subsection has been updated (second paragraph).*

*(2) A new subsection on “Methodological limitations and mitigation strategies” has been added as a separate subsection after “Data analysis”.*

*(3) A new sentence has been added to the existing "Limitations and future research directions" section to briefly acknowledge the technical details of limitations detailed in the methods subsection while keeping the focus on broader implications and future research needs.*

**Reviewer comment 5**: The discussion could be improved by providing a more robust synthesis with the existing literature. As a literature review is not provided, it is difficult for the reader to see ‘significant’ findings, in comparison with what is known in literature. Some discussion is needed to help readers understand how Likert averages can be compared to percentages in the Eurobarometer findings and to speculate on comparisons between student and general population results. 

***Response**: We appreciate the reviewers' suggestion to strengthen literature integration in the Discussion. As noted in response to Comment 1, we have added a Literature Review section that establishes the theoretical context. We have now enhanced the Discussion with additional literature synthesis and methodological clarifications about our comparisons.*

*Regarding the comparison of Likert scale means with Eurobarometer percentages, we acknowledge this methodological challenge and have added explicit clarification of our interpretive approach. We interpret scores above 4.0 as indicating that approximately 80% of respondents lean toward agreement (since 4.0 represents the "agree" point on our scale), while scores around 3.0 suggest neutral or mixed responses. This interpretation allows approximate comparison with Eurobarometer's binary percentage findings, though we explicitly acknowledge these are different measurement approaches. We have also clarified that our student sample represents a specific subpopulation (younger, currently engaged in education) and comparisons with general population surveys should be understood as exploratory rather than definitive.*

*Furthermore, we have integrated additional literature throughout the Discussion to contextualize our findings within established research on environmental attitudes, educational effects on climate concern, and motivational dynamics in recognition systems.*

*Changes made:*

*(1) Added clarification paragraph in Discussion section (after first paragraph)*

*(2) Added methodological justification paragraph (after the above)*

*(3) Enhanced literature integration throughout the Discussion, including: discussion of educational effects on climate concern, analysis of generational differences, education perception gap, and cross-national variations.*

**Reviewer comment 6**: References to the Eurobarometer findings are needed.

***Response***: *We thank the reviewers for noting this. The Eurobarometer references are provided in the manuscript at first mention ('Building on prior European research, such as the Special Eurobarometer 501 (European Commission, 2020a) and 538 (European Commission, 2023)...'). Throughout the Discussion, specific findings are attributed to their source surveys with question numbers (e.g., 'Eurobarometer 538, QC2') to ensure precision while maintaining readability. We believe this approach (full citation followed by abbreviated references with question identifiers) follows standard academic practice while providing complete traceability to specific data points.*

**Reviewer comment 7**: It is unclear why political action is not highlighted in this study; why emphasize individual actions such as eating less meat and asking about government responsibility, without asking whether participants have taken part in climate protests, signed petitions or contacted elected officials?

***Response**: We thank the reviewers for this important observation about political action. This represents a significant limitation of our study design that we now explicitly acknowledge. The OpenPass4Climate survey instrument was developed collaboratively by the project consortium with a primary focus on individual behavioral changes and educational preferences rather than political engagement. This reflects both the project's educational focus and, admittedly, an oversight in not adequately capturing the full spectrum of climate action, particularly collective political engagement that has become increasingly prominent through youth movements like Fridays for Future.*

*While our qualitative data (interviews and focus groups) did capture some political dimensions spontaneously raised by participants—such as the importance of voting for climate-conscious politicians (Portuguese focus group), activism approaches (French focus group), and the role of collective pressure in driving policy change (Spanish focus group)—we acknowledge that the absence of systematic data collection on political action represents a significant gap. This is particularly relevant given that our target demographic (18-24 year-olds) has been at the forefront of climate activism globally.*

*We have added this limitation to our Discussion section and recommend that future research on climate education and recognition systems should explicitly incorporate measures of political engagement, including participation in protests, petition signing, contacting elected officials, and involvement in climate justice movements. This would provide a more complete picture of how educational interventions relate to both individual and collective forms of climate action.*

*Changes made: A new paragraph has been added to the "Limitations and future research directions" subsection to clearly acknowledge the limitation, and a new future research bullet point has been included.*
